# Ameliorated Performance of Sulfonated Poly(Arylene Ether Sulfone) Block Copolymers with Increased Hydrophilic Oligomer Ratio in Proton-Exchange Membrane Fuel Cells Operating at 80% Relative Humidity

**DOI:** 10.3390/polym12091871

**Published:** 2020-08-20

**Authors:** Ae Rhan Kim, Mohanraj Vinothkannan, Kyu Ha Lee, Ji Young Chu, Sumg Kwan Ryu, Hwan Gyu Kim, Jae-Young Lee, Hong-Ki Lee, Dong Jin Yoo

**Affiliations:** 1 Department of Life Science, Jeonbuk National University, Jeonju, Jeollabuk-do 54896, Korea; carumiss@naver.com (K.H.L.); ebbuneg@hanmail.net (J.Y.C.); hgkim@jbnu.ac.kr (H.G.K.); 2 Department of Energy Storage/Conversion Engineering of Graduate School, Hydrogen and Fuel Cell Research Center, Jeonbuk National University, Jeonju, Jeollabuk-do 54896, Korea; hellomong35@naver.com; 3 R&D Education Center for Whole Life Cycle R&D of Fuel Cell Systems, Jeonbuk National University, Jeonju, Jeollabuk-do 54896, Korea; vinothkannanram@gmail.com; 4 Hydrogen Fuel Cell Center, Woosuk University, 151 Dusan-ri, Bongdong-eup, Wanju-gun, Jeollabuk-do 55315, Korea; jjklee@naver.com (J.-Y.L.); hongkil@woosuk.ac.kr (H.-K.L.)

**Keywords:** sulfonated poly(arylene ether sulfone), phase-separation, proton conductivity, proton-exchange membrane fuel cells, 80% relative humidity

## Abstract

We designed and synthesized a series of sulfonated poly(arylene ether sulfone) (SPES) with different hydrophilic or hydrophobic oligomer ratios using poly-condensation strategy. Afterward, we fabricated the corresponding membranes via a solution-casting approach. We verified the SPES membrane chemical structure using nuclear magnetic resonance (^1^H NMR) and confirmed the resulting oligomer ratio. Field-emission scanning electron microscope (FE-SEM) and atomic force microscope (AFM) results revealed that we effectively attained phase separation of the SPES membrane along with an increased hydrophilic oligomer ratio. Thermal stability, glass transition temperature (T_g_) and membrane elongation increased with the ratio of hydrophilic oligomers. SPES membranes with higher hydrophilic oligomer ratios exhibited superior water uptake, ion-exchange capacity, contact angle and water sorption, while retaining reasonable swelling degree. The proton conductivity results showed that SPES containing higher amounts of hydrophilic oligomers provided a 74.7 mS cm^−1^ proton conductivity at 90 °C, which is better than other SPES membranes, but slightly lower than that of Nafion-117 membrane. When integrating SPES membranes with proton-exchange membrane fuel cells (PEMFCs) at 60 °C and 80% relative humidity (RH), the PEMFC power density exhibited a similar increment-pattern like proton conductivity pattern.

## 1. Introduction

Proton-exchange membranes (PEMs) are gaining great attention as both ion conductors and reactant separators for various energy storage/conversion devices such as fuel cells, solar cells and secondary batteries [1,2,3,4]. Among various fuel cells, proton-exchange membrane fuel cells (PEMFCs) have various inherent benefits such as high energy density and efficiency, trivial pollution contributions and quick startup times [5]. Because of these benefits, they are competitive with traditional batteries and combustion engines as clean power sources for automobiles and stationary applications [6,7]. Due to their high ion conductivity and extended chemical stability, perfluorosulfonic acid (PFSA) membranes—especially Nafion—are widely utilized as PEMs for PEMFCs [8,9,10]. PFSA membranes are by no means perfect, however. They are expensive, exhibiting low proton conductivity at high temperatures and low relative humidity (RH) and are highly permeable for fuels. Proton conductivity degradation is caused by Nafion’s inability to retain water in ionic clusters [11]. Moreover, high fuel crossover of Nafion from anode to cathode owing to its direct pathway for fuel permeation [12]. These drawbacks hinder Nafion’s widespread commercialization. Sulfonated poly(arylene ether sulfone) (SPAES) are a hydrocarbon material containing sulfone and ether bridges [13,14]. They are the most commonly adopted material for PEM fabrication because of their design and fabrication flexibility, low price, tunable proton conductivity and film-forming ability [15]. One inherent limitation of the SPES membrane is its low mechanical integrity and, thereby, PEMFC durability. This low mechanical integrity is caused by a high degree of sulfonation or maximum hydrophilic fragment proportion in SPES. A low sulfonation degree or minimal hydrophilic fragment proportion can also be problematic due to decreased water absorption and proton conductivity [15,16]. It is highly desirable to fabricate SPES with equal proportions of hydrophilic and hydrophobic units for PEM applications, since this would offer reasonable ion conductivity, as well as dimensional, thermal and mechanical stability [17,18,19]. In the SPES block copolymer, hydrophilic units provide the ability to conduct protons and uptake water, whereas hydrophobic unit enhance, thermal, mechanical, dimensional and oxidative stabilities of membranes. There are many types of SPES-based block copolymer membranes available that have high ion conductivity, a comprehensive mechanical modulus and thermal stability. While effects of variations in oligomer ratio on mechanical properties, chemical properties and proton conductivity have been studied extensively, there is little understanding of SPES block copolymer membrane suitability for fuel cell application.

Hence, the objective of this study is to prepare a series of SPES block copolymer membranes and apply them in a PEMFC operating under 80% RH and 60 °C. We designed and synthesized (A) hydrophobic oligomer-, (B) hydrophilic oligomer-, (AB) hydrophobic-hydrophilic polymer-, (ABA) hydrophobic-hydrophilic-hydrophobic polymer-, and (BAB) hydrophilic-hydrophobic-hydrophilic polymer-SPES types via a simple and cost-competitive poly condensation strategy. We fabricated the corresponding membranes using a solution-casting approach. We characterized membrane surface morphologies to evaluate phase separation and surface variation for various oligomer ratios. We also investigated water uptake, swelling ratio, ion-exchange capacity, water-contact angle, water sorption and proton conductivity since they are intimately related to fuel-cell performance. Finally, we individually integrated AB, ABA and BAB membranes into the PEMFC device at 80% RH and 60 °C to assess the suitability of these membranes for real-time applications. Among them, the BAB membrane demonstrated a superior PEMFC power output: 124.5 mW cm^−2^ at a 206.3 mA cm^−2^ load-current output.

## 2. Materials and Methods

### 2.1. Materials

Bisphenol-A (BPA, 228.29 g mol^−1^), 4,4’-bis[(4-chlorophenyl) sulfonyl]-1,1’-biphenyl (BCPSBP, 503.42 g mol^−1^), dimethyl sulfoxide (DMSO, 78.13 g mol^−1^), N,N-dimethylacetamide (DMAc, 87.12 g mol^−1^), 4,4’-dichlorobenzophenone (DCBP, 251.11 g mol^−1^), potassium carbonate (K_2_CO_3_, 138.20 g mol^−1^) were supplied by Alfa Aesar, Seoul, South Korea. Other chemicals were procured from Samcheon Chemical Corporation, Seoul, South Korea and used without further purification.

### 2.2. Synthesis of A

We synthesized the A starting with equipment of a nitrogen (N_2_) gas inlet, Dean–Stark condenser and magnetic stirrer with a round-bottomed (RB) flask. In the RB flask, we dispersed BPA (1.50 g, 6.58 mM), DCBP (1.82 g, 7.24 mM) and K_2_CO_3_ (2.00 g, 14.48 mM) in toluene and DMAc at 300 rpm and 140 °C. We increased the temperature to 160 °C and maintained it for 36 h. We recrystallized and filtered the oligomer product using a mixture of water, methanol and acetone (1:1:7 V/V) and dried the product for 24 h at 80 °C. ^1^H NMR (600 MHz, DMSO-d_6_) δ 7.8–7.6, 7.5–7.1, 7.1–7.0, 1.7–1.4; FT-IR, (KBr, cm^−1^) 2967, 1651, 1591, 1496, 1414, 1306, 1277, 1235, 1159, 1104, 1081, 1013, 927, 873, 767, 685, 545, 497.

### 2.3. Synthesis of B

At first, we synthesized a sulfonated BCPSBP (sBCPSBP) according to a previous study[7]. Afterward, we placed the sBCPSBP (6.00 g, 7.80 mM), BPA (1.96 g, 8.58 mM), K_2_CO_3_ (2.40 g, 17.2 mM), toluene and DMAc in an RB flask and proceeded process in the same manner as described for the oligomer A. ^1^H NMR (600 MHz, DMSO-d_6_) δ 8.6–7.2, 7.2–6.6, 1.8–1.4; FT-IR (KBr, cm^−1^) 3448, 3066, 2968, 1585, 1503, 1465, 1307, 1238, 1153, 1116, 1076, 1028, 881, 825, 709, 684, 616, 558.

### 2.4. Synthesis of AB, ABA and BAB

For the synthesis of block copolymers (AB, ABA and BAB), we exploited a poly-condensation strategy. To synthesize AB, A (0.79 g, 2.2 × 10^−5^ M), B (0.31 g, 2.2 × 10^−5^ M) and K_2_CO_3_ (0.04 g, 8.8 × 10^−5^ M) were mixed in 15 mL of toluene and 15 mL of DMAc to the RB flask. For ABA synthesis, A (0.61 g, 4.40 × 10^−5^ M), B (0.79 g, 2.20 × 10^−5^ M) and K_2_CO_3_ (0.04 g, 8.80 × 10^−5^ M) were mixed in 15 mL of toluene and 15 mL of DMAc to the flask. In the case of BAB synthesis, A (0.31 g, 2.2 × 10^−5^ M), B (1.58 g, 4.40 × 10^−5^ M) and K_2_CO_3_ (0.04 g, 8.80 × 10^−5^ M) were mixed in 15 mL of toluene and 15 mL of DMAc to the flask. The rest of the synthesis for AB, ABA and BAB followed the experimental procedure described for the A. AB: ^1^H NMR (600 MHz, DMSO-d_6_) δ 8.5–7.9, 7.8–7.6, 7.6–7.3, 7.3–6.8, 1.8–1.4; FT-IR (KBr, cm^−1^) 3456, 3069, 2967, 1706, 1590, 1498, 1465, 1380, 1305, 1235, 1155, 1122, 1074, 1025, 1013, 927, 876, 831, 766, 683, 614, 557. ABA: ^1^H NMR (600 MHz, DMSO-d_6_) δ 8.5–7.9, 7.8–7.64 7.6–7.4, 7.4–6.8, 1.8–1.4; FT-IR (KBr, cm^−1^) 3518, 3060, 3039, 2966, 2932, 2871, 1710, 1650, 1590, 1496, 1465, 1416, 1306, 1277, 1231, 1157, 1122, 1074, 1013, 927, 873, 767, 683, 614, 558. BAB: ^1^H NMR (600 MHz, DMSO-d_6_) δ 8.5–7.9, 7.8–7.6, 7.6–7.4, 7.4–6.8, 1.8–1.4; FT-IR, (KBr, cm^−1^) 3518, 3060, 2966, 2932, 2871, 1710, 1650, 1590, 1496, 1465, 1416, 1306, 1277, 1231, 1157, 1122, 1074, 1013, 927, 873, 832, 767, 683, 614, 558.

### 2.5. Membrane Preparation

We obtained all the membranes using a simple solvent-casting approach, similar to our previous studies [5,6]. We dissolved block copolymer (AB, ABA or BAB) in DMF solution and stirred for 72 h at 60 °C. Next, we cast the solution on a glass platter and left it to dry for 12 h at 80 °C. Afterward, we pretreated the obtained membranes by submerging them in 1.0 M H_2_SO_4_ for 1 h at 60 °C, followed by rinsing with deionized water. All membrane thicknesses were measured at various random points and found to be ~70 µm.

## 3. Characterizations

We characterized the membranes using a scanning electron microscope (SEM, model: JEOL, JSM-6400, Borken, Germany), atomic force microscope (AFM, model: Bruker, multimode-8, CA, USA), ^1^H nuclear magnetic resonance spectrometer (NMR, model: JNM-ECA-600 MHz, Peabody, St. Louis, MO, USA), Fourier-transform infrared spectroscopy (FT-IR, model: PerkinElmer, MA, USA), thermogravimetric analyzer (TGA, model: Q50, New Castle, DE, USA), differential scanning calorimetry (DSC, model: Q20, New Castle, DE, USA). These instruments are installed at the Center for University-wide Research Facilities (CURF) at Jeonbuk National University (JBNU). We tested 5 × 1 cm (dimension x thickness) membrane mechanical stability using an INSTRON 3365 universal testing machine (UTM, Norwood, MA, USA) at ambient temperature operation. We employed HLC-8320 gel permeation chromatography (GPC, Tokyo, Japan) to explore M_w_, M_n_, M_Z_ and poly dispersity index of the oligomers and polymers. The GPC measurement conditions, such as mobile phase: DMF solvent, detector: RI detector/optional UV detector and column: H type (packed with styrene polymers), were exploited. We determined membrane surface wettability by measuring water-contact angle with a Surface Electro Optics (Model Phoenix-300, Seoul, South Korea) and evaluated water sorption and desorption at different RH via dynamic vapor sorption (DVS, Zurich, Switzerland) on a DVS advantage I measurement system.

We evaluated the membrane water uptake/retention properties over 24 h using each membrane’s weight change before and after water uptake at room temperature. We measured the weights of wet (W_wet_ in grams) and dry (W_dry_ in grams) membrane samples using a Denver four-digit counter balance (Model: S-234) and calculated the water uptake using equation given below [20].

(1) $$\mathrm{Water}\;\mathrm{uptake}\;\left(\%\right)=\;\left[\frac{W_{\mathrm{wet}\;}-W_{\mathrm{dry}}}{W_{\mathrm{dry}}}\right]100$$

Ion-exchange capacity (IEC) helps to evaluate the milliequivalents of ionizable groups exist in membranes. We used a conventional acid–base titration method to measure membrane IEC and the detailed procedure of measurement shown in reference [21]. Equation (2) was used to quantify the IEC.

(2) $$\mathrm{IEC}\;\left(\mathrm{meq}.{\;g}^{-1}\right)=\;\frac{\mathrm{NaOH}\;\mathrm{volume}\;\mathrm{consumed}\times \mathrm{NaOH}\;\mathrm{concentration}}{\mathrm{Dry}\;\mathrm{sample}\;\mathrm{weight}}$$

We measured the membrane’s chemical/oxidative degradation directly using a Fenton reagent solution (2 ppm FeSO_4_ dissolved with 3% hydrogen peroxide). We soaked the membrane (2×2 cm) in the solution for 12 h at room temperature before calculating the residual weight [22].

We tested the membrane’s proton conductivity using alternating current (AC) impedance spectroscopy by integrating approximately 3 cm × 0.5 cm membranes into the four-probe BekkTech cell and measuring conductivity at 40, 50, 60, 70, 80 and 90 °C in 80% RH. We calculated conductivity using Equation (3) [23].

(3) $$\sigma \;\left(\mathrm{mS}/\mathrm{cm}\right)=\frac{L}{\mathrm{RTW}}$$

where *σ* is cation conductivity, L is the length (cm) between the measuring electrodes, R is resistance (Ω), W is film width (cm) and T is film thickness (cm).

Prior to PEMFC analysis, we obtained the membrane electrode assembly (MEA) using a conventional hot-press method [24]. Two commercial catalyst-coated carbon cloths (0.25 mg cm^−2^ Pt loading) sandwiched the AB, ABA, BAB and Nafion-117 membranes individually. Hot-pressing at 70 °C and 80 bar for 2.5 min allowed the catalyst to adhere to the membrane, providing a 5 cm^2^ active area. Afterward, using a hydrogen anode feed and an air cathode feed with 1 and 2 stoichiometries, respectively, we conducted MEA single-cell performance testing at 80% RH and 60 °C. Before testing, MEA conditioning performed at 150-mA cm^−2^ current density for 4 h. We recorded PEMFC H_2_–O_2_ polarization curves using a fuel cell test system (Horizon Fuel Cell Technologies; Model: SKFC-TS001). Each testing polarization point stabilized at the setting current for 3 min and testified the average voltage over the last 25 s.

## 4. Results and Discussion

### 4.1. Synthesis and Structural Properties

**Scheme 1:**
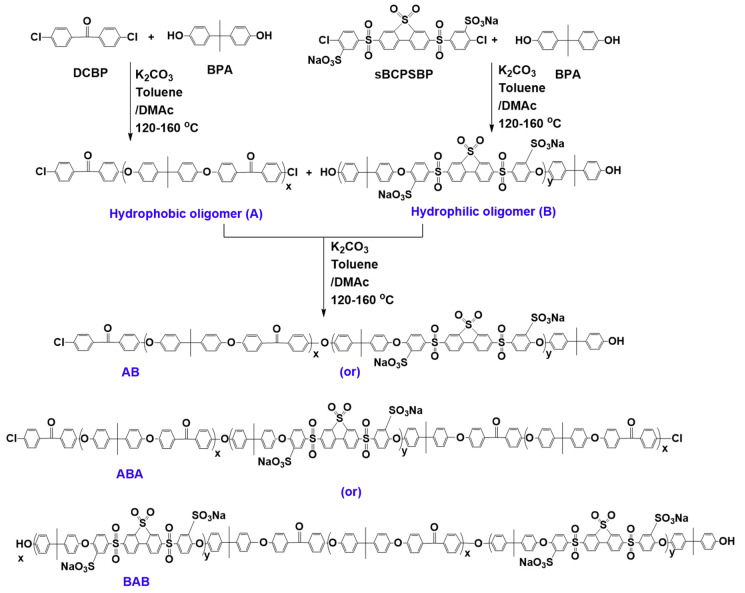
Preparation of the block copolymer.

**Figure 1:**
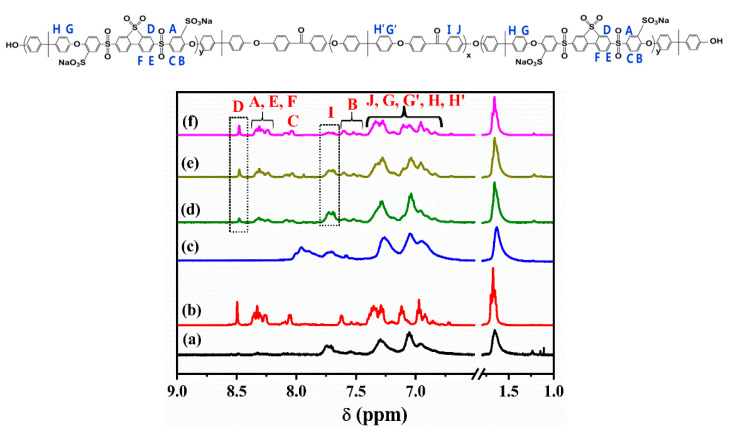
^1^H NMR spectra of (**a**) hydrophobic oligomer (A), (**b**) hydrophilic oligomer (B), (**c**) control polymer, (**d**) hydrophobic-hydrophilic-hydrophobic polymer (ABA), (**e**) hydrophobic-hydrophilic polymer (AB) and (**f**) hydrophilic-hydrophobic-hydrophilic polymer (BAB). Oligomers and polymers were dissolved in DMSO-d_6_ for ^1^H NMR analysis.

**Figure 2:**
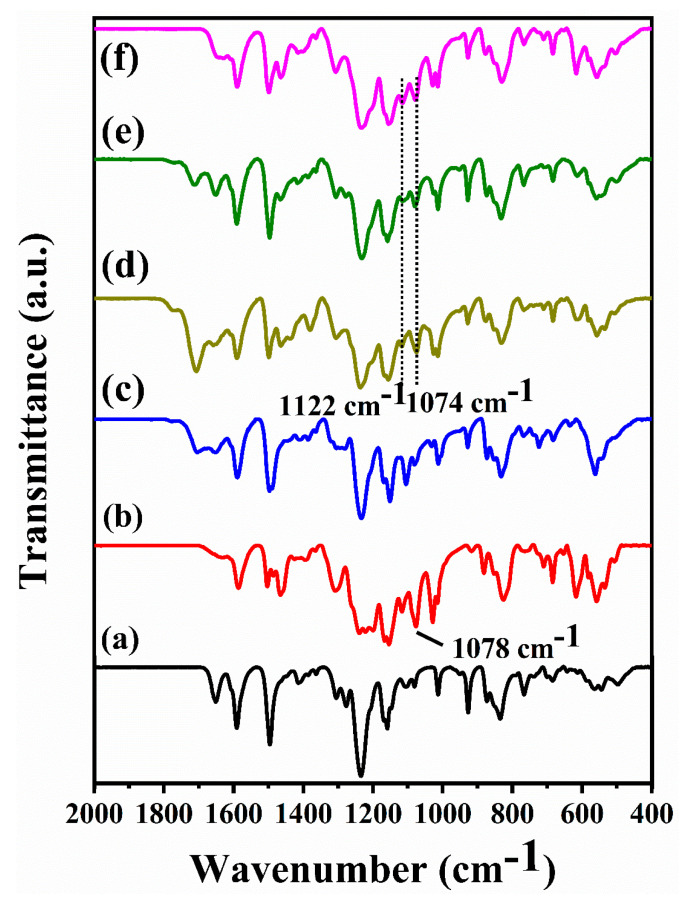
FT-IR spectra of (**a**) A, (**b**) B, (**c**) control polymer, (**d**) AB, (**e**) ABA and (**f**) BAB.

We derived A from DCBP and BPA monomers. We used sBCPSBP and BPA as monomers to synthesize the B. We used the A and B oligomers to synthesize the di-block (AB) and tri-block copolymers (ABA and BAB) (Scheme 1). To determine the chemical structures, we dissolved the hydrophobic oligomer, hydrophilic oligomer, control copolymer, tri-block copolymer (ABA or BAB) and di-block (AB) copolymer in DMSO-d_6_ solvent and analyzed the resulted using NMR spectroscopy (Figure 1). The signals observed between 7.8 and 6.8 ppm were assigned to the aromatic protons of hydrophobic oligomer. The H’ and G’ signals between 7.3 and 6.7 ppm were due to aromatic phenylpropane protons and I signal between 7.8 and 7.66 ppm was from the benzophenone group. The signals between 1.7 and 1.4 ppm were related to the hydrophobic oligomer’s biphenyl propane methyl group. On the other side, NMR spectra of hydrophilic oligomer contained aromatic phenyl ring protons with sulfonate groups. A–F was the lowest signal in the 8.55–7.3 ppm range. Signals between 1.85 and 1.40 ppm confirmed biphenyl propane methyl group presence. The tri-block copolymer (ABA and BAB) and the di-block copolymer (AB) had significant signals in the 8.50 to 6.82 ppm range. These arise from aromatic hydrophobic and hydrophilic proton units. We calculated the degree of sulfonation (DS of hydrophilic oligomer is 50% and used it to synthesize AB, ABA and BAB copolymers. The I signal’s decreasing intensity (7.81–7.62 ppm) from ABA to AB to BAB indicates a hydrophobic oligomer ratio reduction. The D signal’s increasing intensity (8.53–8.45 ppm) from ABA to AB to BAB indicates an incremental polymer hydrophilic ratio increase. A signal in the 7.41–7.16 ppm range indicates a small quantity of non-sulfonated hydrophilic fragments. FT-IR spectra of A, B, control, AB, ABA and BAB membranes are displayed in Figure 2. In the case of copolymers, the absorption band observed at 1013 cm^−1^ was assigned to the stretching vibration of diphenyl ether units. In contrast, the absorption band at 1465 cm^−1^ was related to the stretching vibration of C=C in the polymer backbones. The B, AB, ABA and BAB had the absorption bands at 1122 and 1074 cm^−1^, which were attributed to the sulfonic acid groups. The DCBP, BPA and sBCPSBP monomers were also subjected to ^1^H NMR and FT-IR analysis to confirm their usage during polymer synthesis. The obtained results are given in Supporting information (Figures S1 and S2).

Table 1 shows that A (13,900 g mol^−1^) and B (35,600 g mol^−1^) had considerably high molecular weights. We controlled the A to B ratio at 1:1, 2:1 and 1:2 molar for making AB, ABA and BAB, respectively. The average molecular weights for the di-block and tri-block copolymers (AB, ABA and BAB) were 45,200 g mol^−1^, 61,900 g mol^−1^ and 78,400 g mol^−1^, respectively. The AB, ABA and BAB PDI (M_w_/M_n_) values were 4.6, 6.9 and 6.1, respectively.

### 4.2. Morphologic Behaviors

**Figure 3:**
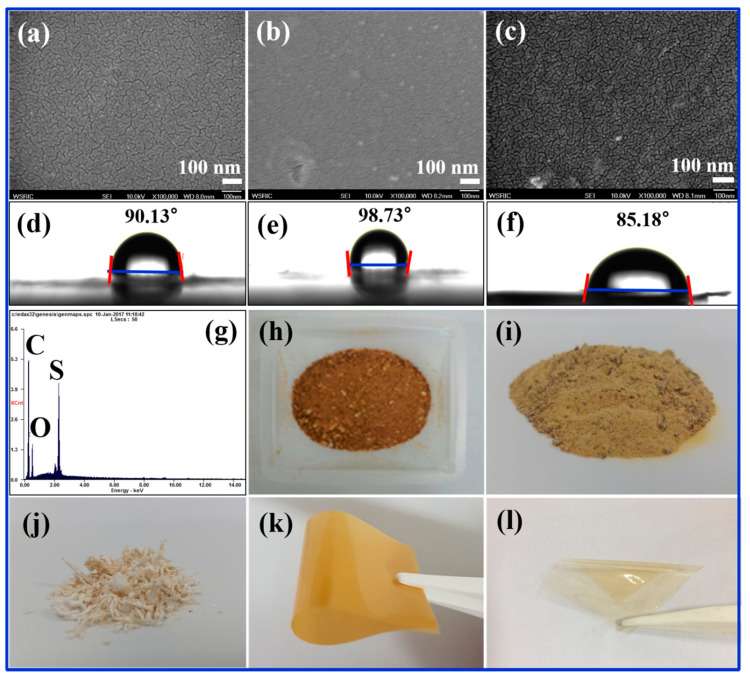
HR-SEM images of (**a**) AB, (**b**) ABA and (**c**) BAB membranes; water-contact angle images to measure the hydrophilicity on membrane surface of (**d**) AB, (**e**) ABA and (**f**) BAB membranes; (**g**) EDX spectra of BAB membrane; digital photographs of (**h**) B, (**i**) A, (**j**) BAB polymer, (**k**) BAB membrane and (**l**) Nafion-117.

**Figure 4:**
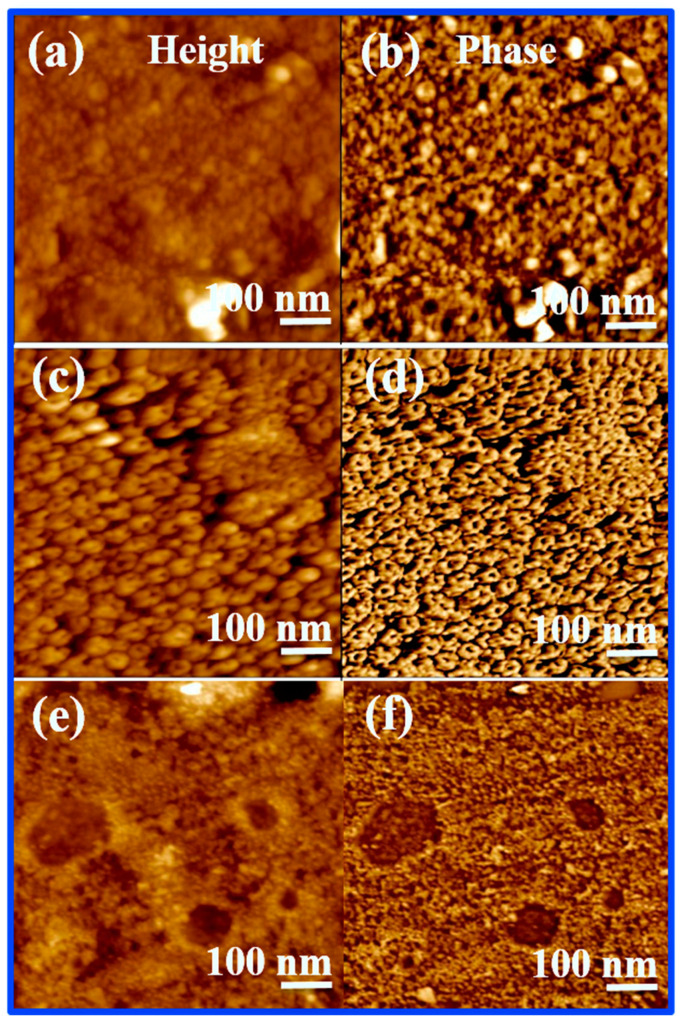
Two-dimensional atomic force microscope (AFM) height images of (**a**) AB, (**c**) ABA and (**e**) BAB membranes; 2D AFM phase images of (**b**) AB, (**d**) ABA and (**f**) BAB membranes.

The FE-SEM morphologies of the as-made AB, ABA and BAB membranes are shown in Figure 3. Each membrane was stained with lead salt before analysis, wherein dark areas exemplify hydrophilic domains containing the lead sulfonate. The BAB membrane exhibits well-developed phase separation with a hydrophilic domain size of ca. 30 nm—as evidenced from Figure 3c. This result is consistent with that of our previous block copolymer containing the same hydrophilic block [15]. Such wide and interconnected hydrophilic domains are beneficial to promote the water contact with membrane surface and facilitate proton conduction in BAB membrane. By contrast, AB (Figure 3a) and ABA (Figure 3b) membranes have only inefficient phase separation, owing to lower hydrophilic blocks in polymer structure [25]. The contact angle of BAB membranes is 85.18° (Figure 3f), representing the presence of hydrophilic surface owing to the availability of polar groups. However, this value is increased to 90.13° and 98.73° in the case of AB and ABA because of lower quantity of hydrophilic unit in membrane. Figure 3h–k show the digital photographs of A, B, BAB polymer and BAB membrane. When compared to A and B, BAB has a fiber form confirming the polymer structure of BAB. The digital photograph of BAB membrane indicate that membrane is being flexible without significant visible defects, like Nafion-117. In AFM (Figure 4), we detected bigger size brighter regions in the ABA membrane (Figure 4c,d) as a result of high ratio of hydrophobic domains. A higher phase-separation quantity for the AB membrane compared to ABA and BAB membranes. This occurs because of the equal hydrophilic and hydrophobic oligomer amounts.

### 4.3. Thermal and Mechanical Behaviors

**Figure 5:**
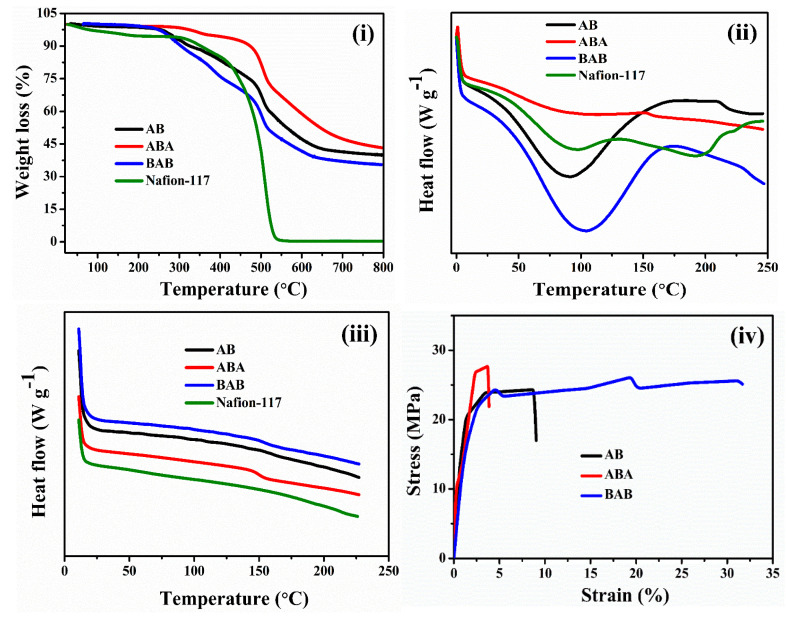
Thermomechanical stabilities of membranes. (**i**) TGA curves; (**ii**) differential scanning calorimetry (DSC) first scan; (**iii**) DSC second; (**iv**) mechanical stability curves of AB, ABA, BAB and Nafion-117 membranes.

Figure 5i exhibits the TGA curves of di-block and tri-block copolymers and Nafion-117 to determine % of weight loss as a function of temperature. Excluding Nafion-117, the other membranes displayed a three stage weight loss: (i) below 160 °C is ascribed to evaporation of water contained in polymer chains, (ii) between 180–390 °C is resulting from split up of sulfonic acid from polymer backbones and (iii) over 450 °C is credited to the deterioration of polymer backbones. At 795 °C, the Nafion-117 membrane measured 99.6% weight loss, possibly due to rapid decomposition of the main skeleton. The ABA membrane had a 56.7% weight loss at the same temperature; the AB membrane had a 60.1% weight loss and BAB had a 64.5% weight loss. These results reveal that the AB and BAB membranes are less thermally stable than the ABA type polymer. The accelerated decomposition of BAB is attributed to the high portion of hydrophilic oligomer in the polymer chains. First scan DSC curves of AB and BAB membranes, except ABA membrane, showed considerable endothermic inflection (T_g_) values (Figure 5ii). All of the membranes had a T_g_ in the 80–150 °C range. Of these membranes, the BAB exhibited the highest T_g_. In general, a membrane’s T_g_ increases when there are more ionic sites in the polymer chain (ionomer effect). A high density of SO_3_H groups increases the membrane’s molecular bulkiness, so T_g_ increases according to bulkiness. This is because these sulfonic acid groups generate a large number of internal hydrogen bonds that restrict polymer chain reorganization during heating and results in increased T_g_ for AB and BAB membranes [26]. For the case of ABA membrane, there is no clear T_g_ found, so, the second scan DSC curves were recorded (Figure 5iii). In the second scan DSC curves, the ABA membrane only exhibits the T_g_ curve, demonstrating the high thermal stability of ABA membrane. All other membranes adopted flat shape curves, suggesting that original structure of polymer were transformed.

Mechanical strength is vital for the membrane materials to function in fuel cells during long-term operation. We evaluated membrane mechanical properties using the stress–strain curves demonstrated in Figure 5iv; the resulting graph is provided in Table 2. Continuous hydrophobic domains with alternative ether and ketone structures acting as a backbone reinforcing material of the membrane, which promote mechanical modulus. AB had a 24.68 MPa tensile strength, 2005.32 MPa Young’s modulus and 9.6% elongation at break. Increasing the hydrophobic oligomer ratio enhanced ABA’s tensile strength to 28.2 MPa and decreased Young’s modulus and elongation at break to 1451.35 MPa and 4.13 MPa, respectively. This suggests that high hydrophobic oligomer ratios cause the ABA membrane to be brittle. The high hydrophilic network structure of BAB concentrated the strain during the stretching process, thereby retaining stress at 24.31 MPa and improving the BAB membrane’s elongation at break to 32.51%. The BAB membrane’s mechanical properties were improved by the high density of sulfonic acid groups causing the membrane to plasticize [27].

### 4.4. Oxidative Stability

**Figure 6:**
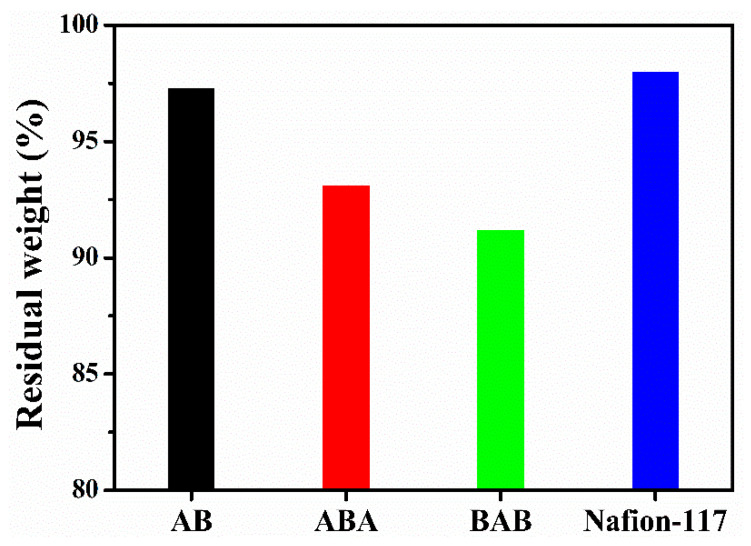
Comparative oxidative stability results of AB, ABA, BAB and Nafion-117 membranes.

We performed the Fenton test to scrutinize the SPAES membranes oxidative stability. After immersing membranes in Fenton’s solution for 12 h, the BAB membrane lost 8% of its original weight, whereas the AB and BAB membranes lost only 6% and 3%, respectively (Figure 6). The higher amounts of OH available in the BAB membrane’s sulfonic acids and phenol groups favor direct free radical reactions (OH• and OOH•) with polymer chains, causing accelerated membrane degradation. Since the AB and ABA membranes have a comparatively low sulfonic acid group density, their degradation is lower.

### 4.5. Water Uptake, Swelling Ratio, IEC and Water Sorption

**Figure 7:**
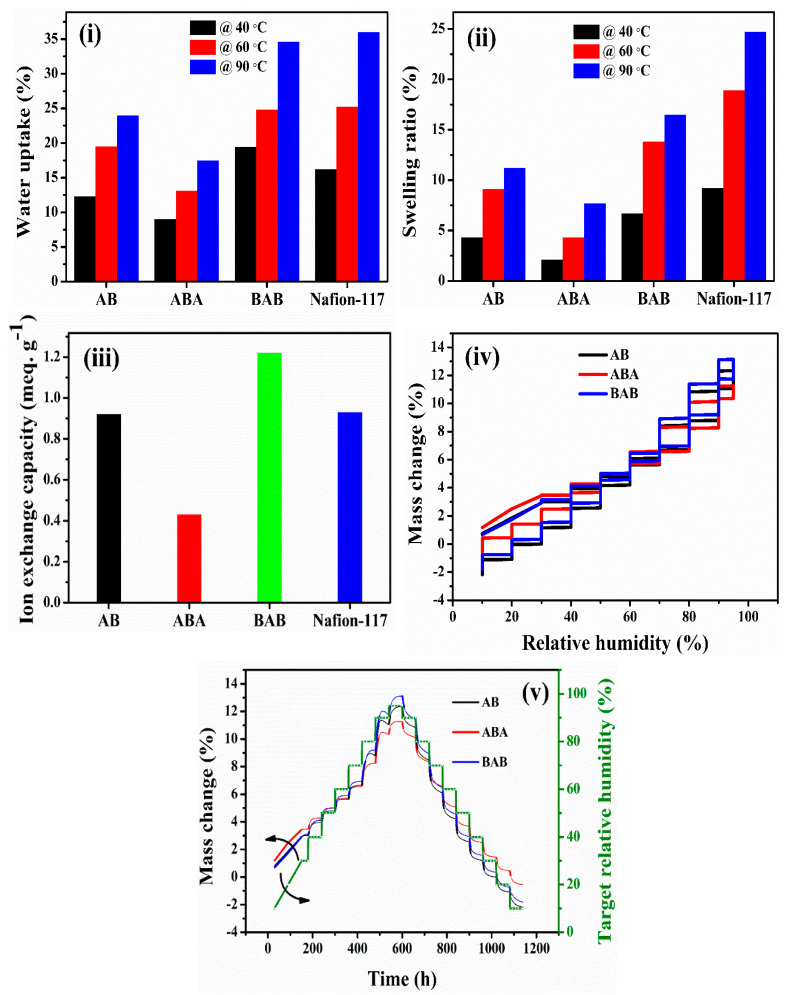
Physiochemical properties of membranes: (**i**) water uptake, (**ii**) swelling ratio, (**iii**) ion-exchange capacity and (**iv**) water sorption properties of AB, ABA, BAB and Nafion-117 membranes.

Figure 7 shows AB, ABA, BAB and Nafion-117 membrane physiochemical characteristics: water uptake, swelling ratio IEC and water sorption. The BAB membrane had a higher water uptake at different temperatures than the AB and ABA membranes because of the high density of sulfonic acid groups and large number of hydroxyl groups present in BAB’s structure (Figure 7i). This led to strong water absorption and retention [28,29]. The membrane’s swelling ratio is dependent to that of water uptake. The maximum swelling ratio was found to be 16.5% at 90 °C for the BAB membrane, which is nearly 1.4- and 2.1-fold higher than those of AB and ABA membranes, respectively (Figure 7ii). The BAB membrane’s high swelling ratio was due to high water uptake in all three dimensions. The BAB membrane also has enhanced IEC as given in Figure 7iii. This is because of the higher sulfonic acid group density in polymer backbones, which facilitates H^+^ the ion mobility. We scrutinized the membrane’s surface hydrophilicity using water sorption measurements and found the BAB membrane’s water sorption to be higher than that for AB and ABA membranes (Figure 7iv,v). The water sorption increased as hydrophilic oligomer ratio increased in polymer chains and is further supported by water-contact angle characteristics, as displayed in Figure 3d–f.

### 4.6. Proton Conductivity and Arrhenius Plots

**Figure 8:**
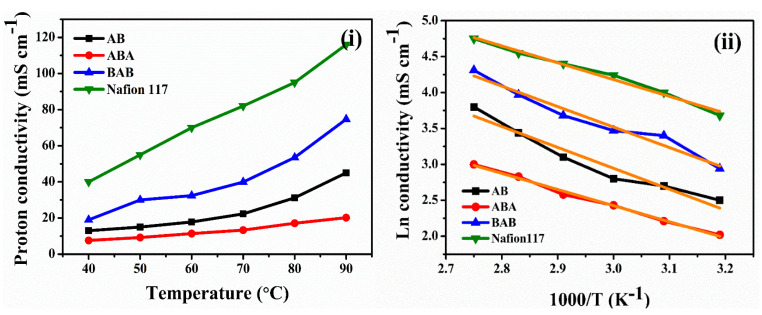
Comparative (**i**) proton conductivity and (**ii**) Arrhenius plots of AB, ABA, BAB and Nafion-117 membranes.

Taking into account the significance of membrane’s proton conductivity during fuel cell operation under slightly low RH, proton conductivity of AB, ABA and BAB membranes was quantified at 80% RH and plotted in Figure 8i. The BAB membrane revealed the conductivities of 19.0, 30.0, 32.4, 39.9, 53.5 and 74.7 mS cm^−1^ at 40, 50, 60, 70, 80 and 90 °C, respectively, while ABA and AB attained relatively lower conductivities at same temperatures. High density of sulfonic acid groups, owing to the maximized proportion of hydrophilic oligomer in BAB membrane, would be responsible for high proton conductivity of BAB membrane. We calculated the activation energy (E_a_) for ion conduction using the Arrhenius plot by Arrhenius formula as provided below.

(4) $$\ln\sigma ={\ln\sigma }_{0}-\frac{E_{a\;}}{\mathrm{RT}}$$

**Scheme 2:**
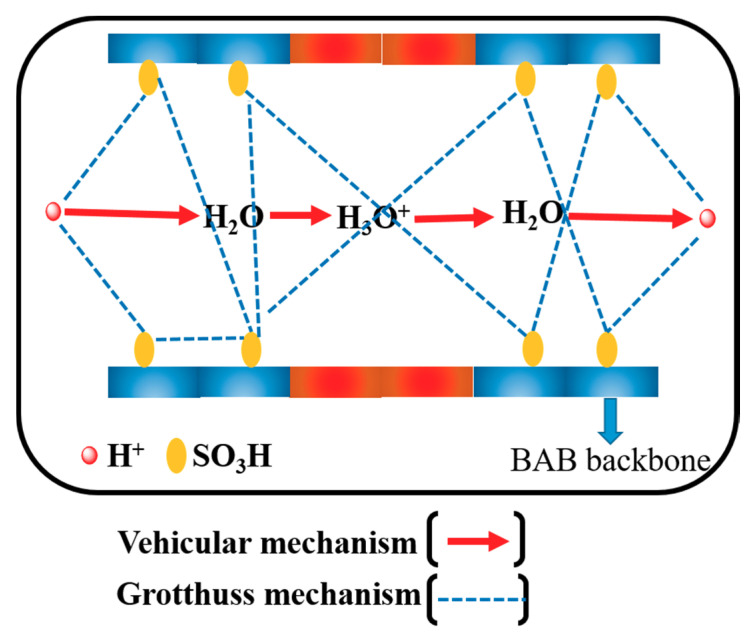
Schematic illustration for proton-transfer mechanisms through BAB polymer structure.

The detailed description about Equation (4) can be found in reference [30,31]. The ABA, AB and BAB membranes revealed E_a_ of 20.5, 18.3 and 16.1 KJ mol^−1^, respectively (Figure 8ii). It is noteworthy that high density of sulfonic acid groups with polymer chains are responsible for good proton conductivity of membrane that assists decrease in E_a_ and bring the BAB membrane suitable for real-time PEMFC technology. The obtained E_a_ values indicate that the membrane follows both vehicular and Grotthuss mechanisms. In the vehicular mechanism, protons are possibly transfer through the water molecules. While, in the Grotthuss mechanism, the protons are hopping through the hydrogen bonding networks between the ionic sites in the polymer chains [32]. Scheme 2 demonstrates the proton-transfer mechanisms through the BAB membrane.

### 4.7. Single Cell Performance

**Figure 9:**
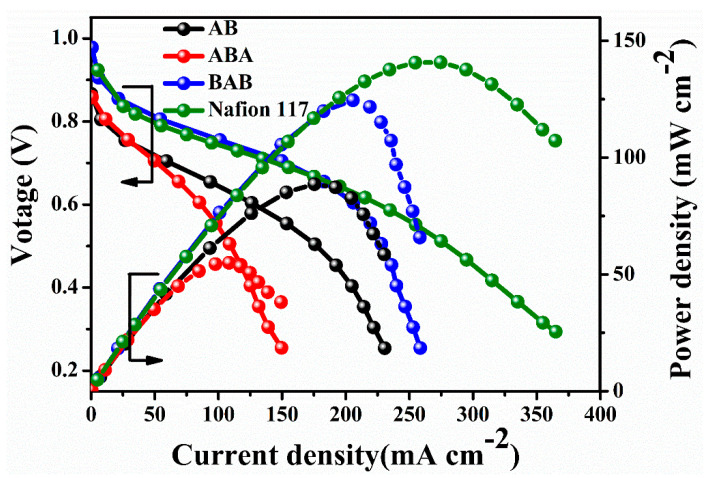
Comparative fuel-cell performance curves of AB, ABA and BAB membranes with Nafion-117 membrane at 60 °C under 80% RH.

We incorporated the AB, ABA, BAB and Nafion-117 membranes into MEAs and measured PEMFCs with H_2_/O_2_ at 60 °C and 80% RH (Figure 9). The AB membrane performed reasonably; the power density reached 88.6 mW cm^−2^ at a 175 mA cm^−2^ current density. As hydrophobic oligomer ratio increased, however, cell performance worsened. The cell power density with an ABA membrane was only 54.9 mW cm^−2^ at a 108.7 mA cm^−2^ current density. This indicates that the ABA membrane’s ohmic impedance and proton conduction decreased dramatically due to decreasing acidic group concentration. By comparison, the BAB membrane’s cell performance remained higher, with a cell power density reaching 124.5 mW cm^−2^ at a 206.3 mA cm^−2^ current density. The enhanced cell performance partly stemmed from good proton conduction, which reduced electrolyte resistance and increased the rate of the cathode reaction. The BAB membrane cell reached a performance level close to that of the Nafion-117 membrane cell (140.8 mW cm^−2^ at a 274.9 mA cm^−2^ current density). This result suggests that the BAB membrane is suitable for potential PEMFC technology.

## 5. Conclusions

In summary—using a facile poly condensation method—we successfully synthesized efficient PEMs based on SPAES block copolymers (AB, ABA and BAB) with varying oligomer ratio for PEMFCs. Compared to AB and ABA membranes, the BAB membrane showed enhanced water uptake, IEC, contact angle and water sorption owing to the higher molar ratio of the hydrophilic oligomer in BAB polymer. Still, BAB membrane exhibited slightly lower physiochemical properties than that of Nafion-117 membrane. On the other side, increasing hydrophilic ratio in BAB membrane slightly reduced the thermal, mechanical and oxidative stabilities of BAB membrane compared to the AB, ABA and Nafion-117 membranes. The BAB membrane’s higher local sulfonic acid concentration caused a microphase separation with opposing hydrophobic domains, which led to increased proton conductivity. The maximum proton conductivity value attained by the BAB membrane was 74.7 mS cm^−1^ at 90 °C under 80% RH. This is a few fold higher than those of AB and ABA membranes and lower than that of Nafion-117 membrane. The BAB membrane’s current and power densities were 258.6 mA cm^−2^ and 124.5 mW cm^−2^, respectively. These are higher values than the AB and ABA membranes at 60 °C and 80% RH, but lower than Nafion-117 membrane. Based on these results, we conclude that the SPAES (BAB) membrane is most suitable for PEMFC applications.

## Figures and Tables

**Table 1:** Molar ratio and molecular weight distributions (M_n_, M_w_, M_z_ and PDI) of the polymers.

Polymer	Molar Ratio ^a^	M_n_, g mol^−1^	M_w_, g mol^−1^	M_z_, g mol^−1^	M_w_/ M_n_ (PDI)
A	10:0	4300	13,900	31,200	3.2
B	0:10	16,500	35,600	68,000	2.1
Control	–	4600	14,800	38,100	3.2
AB	1:1	9600	45,200	212,100	4.6
ABA	2:1	8400	61,900	431,800	6.9
BAB	1:2	12,800	78,400	915,500	6.1

^a^ A:B.

**Table 2:** Mechanical properties of fabricated membranes.

Membrane	Tensile Strength (Mpa)	Elongation at Break (%)	Young’s Modulus (Mpa)
AB	24.68	9.60	2005.32
ABA	28.20	4.13	1451.35
BAB	24.31	32.51	1284

## Supplementary Materials

The following are available online at https://www.mdpi.com/2073-4360/12/9/1871/s1. Figure S1: ^1^H NMR spectra of (a) DCBP, (b) BPA and (c) sBCPSBP. The monomers were dissolved in DMSO-d6 for ^1^H NMR analysis, Figure S2. FT-IR spectra of (a) DCBP, (b) BPA and (c) sBCPSBP.
