# Supplementary material for: Ameliorated Performance of Sulfonated Poly(Arylene Ether Sulfone) Block Copolymers with Increased Hydrophilic Oligomer Ratio in Proton-Exchange Membrane Fuel Cells Operating at 80% Relative Humidity
Source: Polymers (Basel). 2020 Aug 20;12(9):1871. doi: 10.3390/polym12091871 (PMC7563133; doi:10.3390/polym12091871)
Supplement: Supplementary file 1 [file polymers-12-01871-s001.pdf]

Supporting Information

Ameliorated performance of sulfonated poly(arylene ether sulfone) block copolymers with increased hydrophilic oligomer ratio in proton exchange membrane fuel cells operating at 80% relative humidity

Ae Rhan Kim [1, 2,*], Mohanraj Vinothkannan [3], Kyu Ha Lee [1], Ji Young Chu [1], Sumg Kwan Ryu [2], Hwan Gyu Kim [1], Jae-Young Lee [4], Hong-Ki Lee [4] and Dong Jin Yoo [1, 2, 3, **]

[1] Department of Life Science, Jeonbuk National University, Jeonju, Jeollabuk-do, 54896, Republic of Korea

[2] Department of Energy Storage/Conversion Engineering of Graduate School, Hydrogen and Fuel Cell Research Center, Jeonbuk National University, Jeonju, Jeollabuk-do 54896, Republic of Korea

[3] R&D Education Center for Whole Life Cycle R&D of Fuel Cell Systems, Jeonbuk National University, Jeonju, Jeollabuk-do 54896, Republic of Korea

[4] Hydrogen Fuel Cell Center, Woosuk University, 151 Dusan-ri, Bongdong-eup, Wanju-gun, Jeollabuk-do, 55315, Republic of Korea

* Correspondence: aerhankim@jbnu.ac.kr (A.R. Kim); djyoo@jbnu.ac.kr (D.J. Yoo)

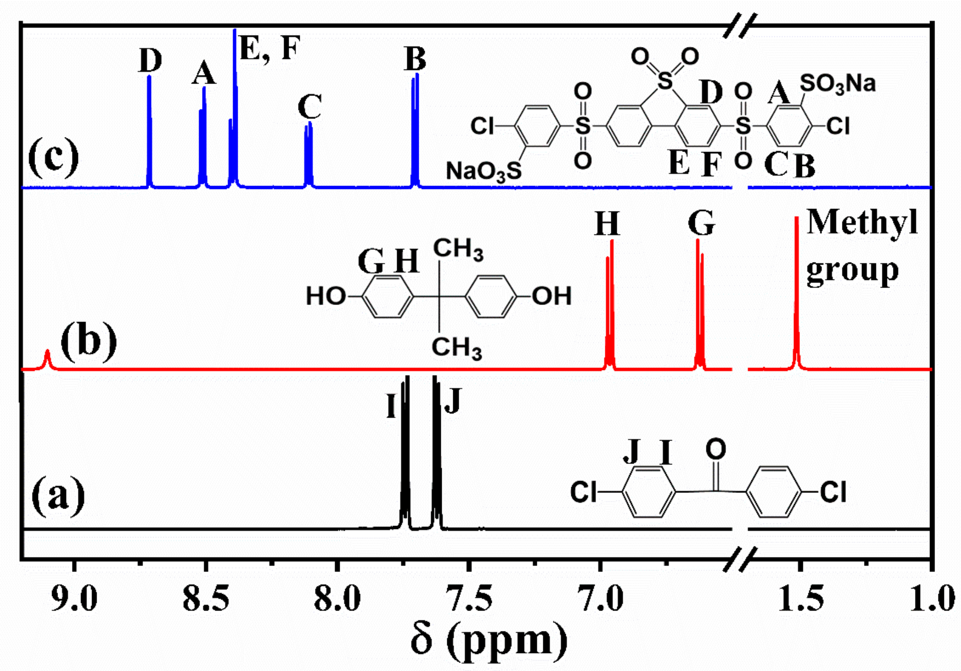

Figure S1. ^{1}H NMR spectra of (a) DCBP, (b) BPA and (c) sBCPSBP. The monomers were dissolved in DMSO-d_6 for ^{1}H NMR analysis.

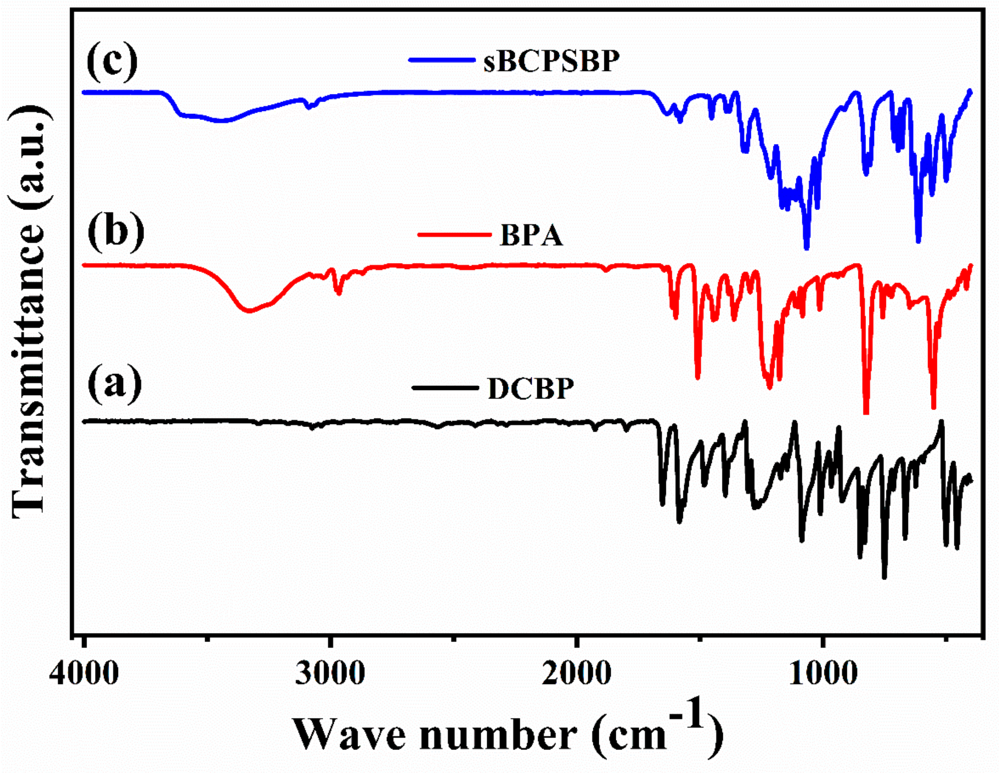

Figure S2. FT-IR spectra of (a) DCBP, (b) BPA and (c) sBCPSBP.
